# Supplementary material for: Mutation and Microsatellite Instability (MSI) Affect the Differential Gene Expression of Folic Acid and 5-Flourouracil Metabolism-Related Genes in Colorectal Carcinoma
Source: Curr Oncol. 2025 Nov 26;32(12):661. doi: 10.3390/curroncol32120661 (PMC12732240; doi:10.3390/curroncol32120661)
Supplement: Supplementary file 1 [file curroncol-32-00661-s001.zip › Supplementary Figure S1.pdf]

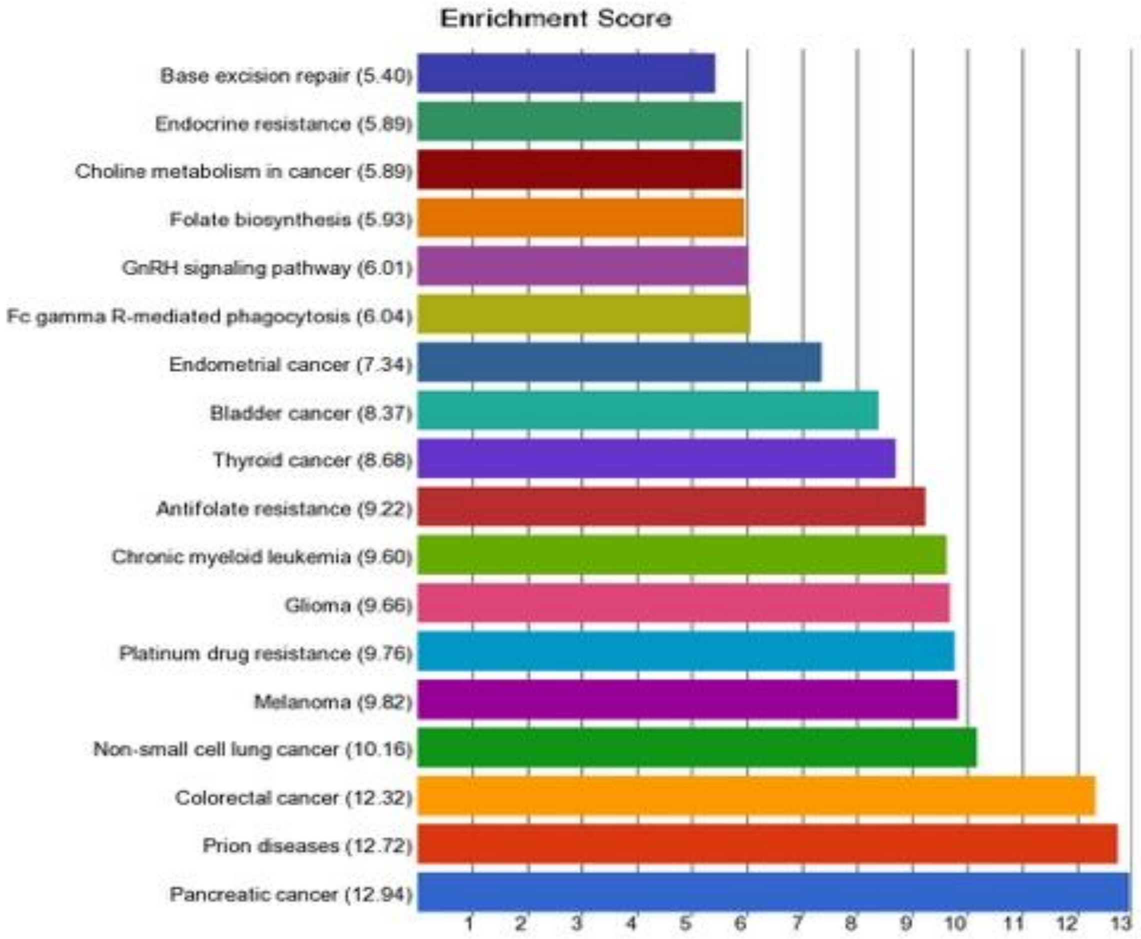

Supplementary Figure S1. Gene set enrichment analysis of the pathways that involve 29 folic acid related genes that were significantly influenced by age of onset.
