# Supplementary material for: Mutation and Microsatellite Instability (MSI) Affect the Differential Gene Expression of Folic Acid and 5-Flourouracil Metabolism-Related Genes in Colorectal Carcinoma
Source: Curr Oncol. 2025 Nov 26;32(12):661. doi: 10.3390/curroncol32120661 (PMC12732240; doi:10.3390/curroncol32120661)
Supplement: Supplementary file 1 [file curroncol-32-00661-s001.zip › Supplementary Table S3.pdf]

Table S3. Significant Paired Analysis Results (FC at least 1.2 in either direction at FDR 0.05) from 380 Probes Covering 180 Genes.

Probe set ID	Name of gene	Fold-Change	(95% CI)	p-value	FDR p-value
ILMN_1673711	HSP90AB1	1.78087	1.66 - 1.91	1.26×10^{-24}	3.68×10^{-22}
ILMN_1800317	WNT5A	2.50354	2.23 - 2.81	2.24E-24	3.68E-22
ILMN_1723971	SLC29A1	1.97914	1.80 - 2.17	1.96E-22	2.10E-20
ILMN_1765966	CHGB	-3.27852	-3.85 - -2.79	2.56E-22	2.10E-20
ILMN_1689001	CDK4	1.7849	1.65 - 1.93	3.60E-22	2.36E-20
ILMN_2338963	SLC29A1	1.7246	1.60 - 1.86	1.50E-21	8.21E-20
ILMN_2246956	BCL2	-1.8574	-2.04 - -1.69	2.26E-20	1.06E-18
ILMN_1803180	PRDX6	-1.85444	-2.05 - -1.68	6.50E-19	2.67E-17
ILMN_1801119	BCL2	-1.71613	-1.87 - -1.57	1.71E-18	6.25E-17
ILMN_1686367	HSPA8	1.43925	1.35 - 1.53	8.32E-18	2.73E-16
ILMN_1720373	SLC7A5	2.68475	2.26 - 3.19	2.69E-17	8.03E-16
ILMN_2366388	PRDX1	1.50809	1.40 - 1.62	4.02E-17	1.10E-15
ILMN_2393573	RASSF1	1.292	1.23 - 1.35	1.14E-16	2.87E-15
ILMN_1734205	RASSF1	1.34999	1.28 - 1.43	3.16E-16	7.40E-15
ILMN_1788166	TTK	1.54609	1.43 - 1.68	4.47E-16	9.77E-15
ILMN_2366391	PRDX1	1.51496	1.40 - 1.64	4.86E-16	9.96E-15
ILMN_1802404	ABCC1	1.32038	1.25 - 1.39	6.67E-16	1.29E-14
ILMN_1688480	CCND1	1.94754	1.72 - 2.21	1.01E-15	1.83E-14
ILMN_1725260	CDC25C	1.36668	1.29 - 1.45	1.06E-15	1.83E-14
ILMN_1808132	FAS	-1.27807	-1.34 - -1.22	1.74E-15	2.85E-14
ILMN_2202948	BUB1	1.66048	1.51 - 1.83	2.17E-15	3.39E-14
ILMN_2373763	CASP7	-1.66858	-1.84 - -1.51	2.54E-15	3.78E-14
ILMN_1680955	AURKA	1.79636	1.60 - 2.01	4.26E-15	6.07E-14
ILMN_1747911	CDC2	1.83001	1.62 - 2.06	5.68E-15	7.76E-14
ILMN_2349459	BIRC5	1.69672	1.53 - 1.88	7.18E-15	9.42E-14

ILMN_1801307	TNFSF10	-1.69826	-1.89 - -1.53	7.89E-15	9.95E-14
ILMN_1664630	CHEK1	1.64626	1.49 - 1.82	8.79E-15	1.07E-13
ILMN_1660436	HSPA1B	1.89159	1.66 - 2.15	1.17E-14	1.38E-13
ILMN_2319077	FAS	-1.50069	-1.63 - -1.38	1.27E-14	1.44E-13
ILMN_2357438	AURKA	1.81687	1.61 - 2.05	1.58E-14	1.72E-13
ILMN_1672496	DNAJA1	1.45756	1.35 - 1.57	2.80E-14	2.97E-13
ILMN_1775304	DNAJB1	1.52127	1.39 - 1.66	4.67E-14	4.78E-13
ILMN_2413084	HSPA8	1.43912	1.33 - 1.55	8.79E-14	8.74E-13
ILMN_1710428	CDC2	1.38367	1.29 - 1.48	1.70E-13	1.64E-12
ILMN_2336781	SOD2	2.05878	1.76 - 2.41	1.93E-13	1.80E-12
ILMN_1667260	MAPK3	-1.69561	-1.90 - -1.51	2.22E-13	2.02E-12
ILMN_2402341	MAPK3	-1.662	-1.86 - -1.49	2.54E-13	2.25E-12
ILMN_1768662	UCK2	1.31399	1.24 - 1.39	2.64E-13	2.28E-12
ILMN_2374159	HERPUD1	-1.55379	-1.71 - -1.41	4.50E-13	3.78E-12
ILMN_1654118	BCL2L1	1.52815	1.39 - 1.68	5.03E-13	4.12E-12
ILMN_2373515	HSP90AA1	1.34465	1.26 - 1.44	5.41E-13	4.33E-12
ILMN_1777564	MAD2L1	1.78874	1.56 - 2.05	5.11E-12	3.96E-11
ILMN_2406501	SOD2	1.75456	1.54 - 2.00	5.19E-12	3.96E-11
ILMN_1710756	ENO1	1.39373	1.29 - 1.51	5.96E-12	4.44E-11
ILMN_1667213	DFFA	1.21417	1.16 - 1.27	8.07E-12	5.88E-11
ILMN_2407619	CDC25C	1.31453	1.23 - 1.40	1.08E-11	7.67E-11
ILMN_1803124	BIRC5	1.229	1.17 - 1.29	1.41E-11	9.82E-11
ILMN_1691097	HSP90AA1	1.59179	1.42 - 1.79	2.67E-11	1.82E-10
ILMN_1729051	MSH6	1.39517	1.28 - 1.52	3.12E-11	2.09E-10
ILMN_2374164	HERPUD1	-1.5329	-1.71 - -1.38	3.56E-11	2.34E-10
ILMN_1719696	PLD1	-1.43397	-1.57 - -1.31	4.23E-11	2.72E-10
ILMN_1749662	GPX1	1.30883	1.22 - 1.40	5.80E-11	3.66E-10
ILMN_1791280	HSPB8	-1.34179	-1.45 - -1.24	6.52E-11	4.03E-10

ILMN_1712803	CCNB1	1.42364	1.30 - 1.56	8.20E-11	4.89E-10
ILMN_1701134	PTEN	-1.20776	-1.27 - -1.15	9.49E-11	5.56E-10
ILMN_2222234	PRDX4	1.36535	1.26 - 1.48	1.50E-10	8.66E-10
ILMN_1734830	MTHFR	-1.20271	-1.26 - -1.15	1.57E-10	8.90E-10
ILMN_1663390	CDC20	1.69913	1.48 - 1.95	1.86E-10	1.03E-09
ILMN_1736176	PLK1	1.24075	1.17 - 1.31	2.19E-10	1.18E-09
ILMN_1667476	LTBR	1.29694	1.21 - 1.39	3.43E-10	1.81E-09
ILMN_1730416	CYCS	-1.45467	-1.61 - -1.31	4.08E-10	2.13E-09
ILMN_2395236	CHEK2	1.20755	1.15 - 1.27	4.37E-10	2.24E-09
ILMN_1795715	DPYD	-1.23998	-1.31 - -1.17	4.84E-10	2.43E-09
ILMN_1757350	CTNNB1	1.50017	1.34 - 1.68	4.88E-10	2.43E-09
ILMN_1742410	BCL2L1	1.22753	1.16 - 1.30	1.11E-09	5.35E-09
ILMN_2362974	CASP7	-1.32139	-1.43 - -1.22	1.32E-09	6.26E-09
ILMN_1779356	TP53	-1.21082	-1.28 - -1.15	1.39E-09	6.53E-09
ILMN_1684217	AURKB	1.41434	1.28 - 1.56	1.80E-09	8.32E-09
ILMN_1746396	CTNNB1	1.46362	1.31 - 1.63	1.90E-09	8.66E-09
ILMN_2311089	BRCA1	1.38491	1.26 - 1.52	1.97E-09	8.83E-09
ILMN_2331010	TNFRSF10B	1.22834	1.16 - 1.30	2.97E-09	1.32E-08
ILMN_2321064	BAX	1.25597	1.17 - 1.35	2.23E-08	9.39E-08
ILMN_1789641	ABCG2	-1.26165	-1.36 - -1.17	2.74E-08	1.14E-07
ILMN_1717714	CDKN2A	1.23182	1.15 - 1.32	2.90E-08	1.19E-07
ILMN_1676984	DDIT3	1.25948	1.17 - 1.36	3.29E-08	1.32E-07
ILMN_1738027	BRCA1	1.24487	1.16 - 1.34	5.06E-08	1.98E-07
ILMN_1717056	TXNRD1	1.21855	1.14 - 1.30	6.29E-08	2.43E-07
ILMN_1707720	SLC1A5	1.31458	1.20 - 1.44	1.00E-07	3.73E-07
ILMN_1699265	TNFRSF10B	1.32058	1.20 - 1.45	1.19E-07	4.39E-07
ILMN_1688698	ZEB2	-1.2335	-1.32 - -1.15	1.33E-07	4.85E-07
ILMN_1678669	RRM2	1.27833	1.18 - 1.39	1.43E-07	5.16E-07

ILMN_1784602	CDKN1A	-1.42697	-1.61 - -1.26	1.92E-07	6.78E-07
ILMN_1812070	ABCB1	-1.70959	-2.06 - -1.42	2.00E-07	6.99E-07
ILMN_2370825	ATM	-1.27237	-1.39 - -1.17	4.37E-07	1.48E-06
ILMN_1700606	RNF43	1.66331	1.39 - 2.00	5.64E-07	1.89E-06
ILMN_1787461	RUNX3	-1.37375	-1.54 - -1.22	6.17E-07	2.01E-06
ILMN_1734353	GPX4	1.25833	1.16 - 1.37	6.19E-07	2.01E-06
ILMN_1706386	SLC39A4	1.26647	1.16 - 1.38	7.43E-07	2.37E-06
ILMN_1779214	ATM	-1.24556	-1.35 - -1.15	8.73E-07	2.75E-06
ILMN_1693145	BUB3	1.20414	1.12 - 1.29	9.42E-07	2.94E-06
ILMN_2358457	ATF4	1.23045	1.14 - 1.33	1.55E-06	4.74E-06
ILMN_2378952	GPX4	1.23771	1.14 - 1.34	1.73E-06	5.26E-06
ILMN_1745806	PEMT	1.2187	1.13 - 1.31	2.03E-06	6.12E-06
ILMN_1661886	APEX1	1.2093	1.12 - 1.30	2.82E-06	8.42E-06
ILMN_2395240	CHEK2	1.21346	1.12 - 1.31	3.96E-06	1.17E-05
ILMN_2094875	ABCB1	-1.5083	-1.78 - -1.28	4.14E-06	1.21E-05
ILMN_2346339	FOLR1	1.54334	1.29 - 1.85	1.20E-05	3.34E-05
ILMN_1661733	FOLR1	1.51489	1.25 - 1.83	4.54E-05	0.000121
ILMN_1686871	PARP1	1.21346	1.11 - 1.33	4.70E-05	0.000124
ILMN_1806040	TYMS	1.3108	1.15 - 1.49	8.75E-05	0.000224
ILMN_1674236	HSPB1	1.28369	1.13 - 1.46	0.000273	0.000644
ILMN_1778561	WEE1	1.20575	1.09 - 1.33	0.000389	0.000871
ILMN_1724480	AXIN2	1.50477	1.20 - 1.89	0.000637	0.001374
ILMN_1720282	NQO1	1.33776	1.12 - 1.60	0.002169	0.004391
ILMN_1804735	CBS	1.30833	1.10 - 1.55	0.002567	0.005103
