# Supplementary material for: Mutation and Microsatellite Instability (MSI) Affect the Differential Gene Expression of Folic Acid and 5-Flourouracil Metabolism-Related Genes in Colorectal Carcinoma
Source: Curr Oncol. 2025 Nov 26;32(12):661. doi: 10.3390/curroncol32120661 (PMC12732240; doi:10.3390/curroncol32120661)
Supplement: Supplementary file 1 [file curroncol-32-00661-s001.zip › Supplementary Table S4-S11.pdf]

Supplementary Tables

Table S4. Pathways with significant differential expression in relation to age of onset.

**Gene Set**	**Interaction *p***	**Interaction FDR p**	**Late-Onset (>40 Years)**		**Early-Onset (<40 Years)**	
			**Fold Change**	**(95% CI)**	**Fold Change**	**(95% CI)**
Cell Cycle Checkpoint Genes	2.45×10^{-4}	1.72×10^{-3}	1.29	(1.27 to 1.32)	1.21	(1.19 to 1.24)
Oxidative Stress Response Genes	1.56×10^{-2}	5.68×10^{-2}	1.12	(1.1 to 1.15)	1.07	(1.04 to 1.10)
Apoptotic gene	2.83×10^{-2}	7.92×10^{-2}	-1.02	(-1.03 to -1.01)	-1.04	(-1.05 to -1.02)
DNA Repair Mechanisms	4.52×10^{-2}	1.05×10^{-1}	1.05	(1.03 to 1.06)	1.04	(1.02 to 1.06)

Table S5. Pathways with significant differential expression in relation to grade.

Gene Set	Interaction *p*	Interaction FDR p	High Grade		Low Grade	
			Fold Change	(95% CI)	Fold Change	(95% CI)
Cell Cycle Checkpoint Genes	2.59×10^{-16}	3.62×10^{-15}	1.15	(1.11 to 1.19)	1.29	(1.27 to 1.31)
Oxidative Stress Response Genes	1.89×10^{-3}	3.31×10^{-3}	1.05	(1.01 to 1.09)	1.12	(1.10 to 1.14)

Table S6. Pathways with significant differential expression in relation to PN Invasion.

Gene Set	Interaction p	Interaction FDR p	No PN Invasion		PN Invasion Present	
			Fold Change	(95% CI)	Fold Change	(95% CI)
Cell Cycle Checkpoint Genes	8.36×10^{-8}	1.17×10^{-6}	1.28	(1.26 to 1.30)	1.2	(1.16 to 1.24)
Oxidative Stress Response Genes	1.25×10^{-3}	8.72×10^{-3}	1.12	(1.10 to 1.14)	1.05	(1.01 to 1.09)
Signaling pathway	3.44×10^{-3}	1.61×10^{-2}	1.09	(1.07 to 1.11)	1.05	(1.01 to 1.09)
Apoptotic gene	9.17×10^{-3}	3.21×10^{-2}	-1.02	(-1.03 to -1.01)	-1.04	(-1.07 to -1.02)

Table S7. Pathways with significant differential expression in relation to sex.

Gene Set	Interaction p	Interaction FDR p	Female		Male	
			Fold Change	(95% CI)	Fold Change	(95% CI)
Oxidative Stress Response Genes	5.21×10^{-5}	7.29×10^{-4}	1.09	(1.06 to 1.12)	1.11	(1.09 to 1.14)
Drug Transport	3.03×10^{-4}	2.12×10^{-3}	1.1	(1.06 to 1.13)	1.06	(1.03 to 1.09)
Apoptotic gene	1.54×10^{-3}	5.38×10^{-3}	-1.04	(-1.06 to -1.03)	-1.03	(-1.03 to -1.00)
Cell Cycle Checkpoint Genes	3.90×10^{-2}	9.14×10^{-2}	1.29	(1.26 to 1.32)	1.25	(1.22 to 1.27)
Heat Shock Response Genes	3.92×10^{-2}	9.14×10^{-2}	1.35	(1.28 to 1.43)	1.28	(1.23 to 1.34)

Table S8. Pathways with significant differential expression in relation to signet ring presence.

Gene Set	Interaction p	Interaction FDR p	Signet Absent		Signet Present	
			Fold Change	(95% CI)	Fold Change	(95% CI)
Cell Cycle Checkpoint Genes	1.68×10^{-17}	2.35×10^{-16}	1.29	(1.27 to 1.31)	1.19	(1.16 to 1.22)
EMT & tumor invasion	5.11×10^{-7}	3.57×10^{-6}	-1.05	(-1.10 to -1.00)	1.14	(1.07 to 1.23)
Oxidative Stress Response Genes	2.52×10^{-3}	1.18×10^{-2}	1.12	(1.10 to 1.14)	1.06	(1.03 to 1.10)

Table S9. Pathways with significant differential expression in relation to CRC stage.

Gene Set	Interaction p	Interaction FDR p	Stage 1		Stage 2		Stage 3	
			Fold Change	(95% CI)	Fold Change	(95% CI)	Fold Change	(95% CI)
Cell Cycle Checkpoint Genes	9.64×10^{-6}	1.35×10^{-4}	1.34	(1.30 to 1.39)	1.27	(1.23 to 1.30)	1.24	(1.21 to 1.26)
Heat Shock Response Genes	1.82×10^{-4}	1.27×10^{-3}	1.24	(1.14 to 1.35)	1.26	(1.19 to 1.34)	1.36	(1.30 to 1.43)
Oxidative Stress Response Genes	2.40×10^{-3}	1.11×10^{-2}	1.16	(1.12 to 1.21)	1.09	(1.06 to 1.12)	1.09	(1.07 to 1.12)
Folate & One carbon metabolism	1.26×10^{-2}	3.52×10^{-2}	1.08	(1.05 to 1.12)	1.04	(1.01 to 1.06)	1.03	(1.01 to 1.05)

Table S10. Pathways with significant differential expression in relation telomere status.

Gene Set	Interaction p	Interaction FDR p	Pts with no TEL Shortening		Pts with TEL Shortening	
			Fold Change	(95% CI)	Fold Change	(95% CI)
Cell Cycle Checkpoint Genes	4.14×10^{-6}	5.80×10^{-5}	1.20	(1.16 to 1.23)	1.29	(1.27 to 1.31)

Table S11. Pathways with significant differential expression in relation to TIL status.

Gene Set	Interaction p	Interaction FDR p	TIL Binary 0		TIL Binary 1	
			Fold Change	(95% CI)	Fold Change	(95% CI)
Oxidative Stress Response Genes	3.42×10^{-3}	3.73×10^{-2}	1.07	(1.05 to 1.10)	1.14	(1.11 to 1.17)
Signaling pathway	5.34×10^{-3}	3.73×10^{-2}	1.05	(1.03 to 1.08)	1.11	(1.08 to 1.13)
