# Supplementary material for: Mutation and Microsatellite Instability (MSI) Affect the Differential Gene Expression of Folic Acid and 5-Flourouracil Metabolism-Related Genes in Colorectal Carcinoma
Source: Curr Oncol. 2025 Nov 26;32(12):661. doi: 10.3390/curroncol32120661 (PMC12732240; doi:10.3390/curroncol32120661)
Supplement: Supplementary file 1 [file curroncol-32-00661-s001.zip › Supplementary Table S12.pdf]

Table S12. Differential expression of 29 folic acid related genes in early vs. late onset CRC.

Gene	Interaction p-value	FDR (p-value	(CRC * Young vs. Normal * Young)			(CRC * Old vs. Normal * Old)		
			Fold Change	(95% CI)	p-value	Fold-Change	(95% CI)	p-value
MSH2	0.000145	0.048135	-1.00	(-1.06-1.06)	0.973312	1.10	(1.05-1.15)	0.000116
PLD1	0.000908	0.150197	-1.27	(-1.45- -1.11)	0.00051	-1.52	(-1.69- -1.38)	9.17E-14
PARP2	0.003083	0.340198	1.1	(1.02-1.19)	0.016242	1.23	(1.16-1.30)	2.14E-10
DFFA	0.005436	0.354128	1.13	(1.05-1.21)	0.00059	1.26	(1.2-1.33)	2.80E-15
XBP1	0.00933	0.354128	1.03	(-1.23-1.29)	0.826121	-1.19	(-1.42- -1.00)	0.048719
ATF6	0.009611	0.354128	1.08	(-1.01-1.19)	0.098944	1.16	(1.07-1.25)	0.000141
BIRC5	0.010815	0.354128	1.12	(1.03-1.20)	0.005989	1.29	(1.21-1.37)	1.19E-13
APEX1	0.011255	0.354128	1.06	(-1.06-1.20)	0.331988	1.27	(1.15-1.39)	2.37E-06
MAPK3	0.011561	0.354128	-1.40	(-1.66- -1.17)	0.000238	-1.84	(-2.11- -1.61)	1.26E-15
HSPA5	0.011589	0.354128	-1.06	(-1.22-1.08)	0.377135	-1.05	(-1.17-1.05)	0.316404
XBP1	0.012036	0.354128	1.01	(-1.26-1.28)	0.953177	-1.19	(-1.43-1.00)	0.058312
MAPK3	0.012839	0.354128	-1.42	(-1.68- -1.20)	6.77E-05	-1.78	(-2.03- -1.57)	2.96E-15
CSNK1A1	0.018355	0.436603	-1.02	(-1.10-1.04)	0.507254	1.11	(1.05-1.17)	0.000294
FPGS	0.019686	0.436603	1.02	(-1.03-1.07)	0.465476	1.10	(1.05-1.15)	1.35E-05
BIRC5	0.022032	0.436603	1.50	(1.25-1.79)	1.75E-05	1.84	(1.60-2.11)	6.15E-15
ENO1	0.022478	0.436603	1.22	(1.07-1.38)	0.002575	1.52	(1.38-1.67)	2.22E-14
CHGB	0.02305	0.436603	-3.11	(-4.02- -2.40)	6.85E-15	-3.28	(-3.40- -2.70)	4.53E-23
MAPK1	0.025159	0.436603	1.02	(-1.07-1.12)	0.617834	-1.00	(-1.08- -1.07)	0.929933
GGH	0.025746	0.436603	-1.07	(-1.32-1.14)	0.495128	1.21	(1.03-1.42)	0.017899
GPX4	0.027573	0.436603	1.17	(1.03-1.33)	0.017687	1.28	(1.16-1.41)	3.14E-06
UPP1	0.027713	0.436603	1.00	(-1.05-1.05)	0.919818	-1.00	(-1.04-1.04)	0.997523
ERCC5	0.029019	0.436603	1.05	(-1.10-1.20)	0.523637	-1.22	(-1.36- -1.09)	0.00045
APEX1	0.03137	0.451448	1.02	(-1.14-1.19)	0.759785	1.26	(1.12-1.41)	0.000142
PARP2	0.03409	0.47016	1.12	(1.03-1.22)	0.005591	1.19	(1.12-1.26)	2.58E-07
ABCC5	0.038767	0.513278	1.11	(-1.03-1.27)	0.115653	-1.12	(-1.24- -1.01)	0.034003
TNFSF10	0.045585	0.561097	-1.56	(-1.90- -1.28)	9.92E-06	-1.77	-2.05- -1.53)	2.47E-12
GADD45A	0.047019	0.561097	-1.22	(-1.45- -1.02)	0.023313	-1.04	(-1.19-1.09)	0.530332
CDK4	0.047882	0.561097	1.57	(1.38-1.78)	6.58E-11	1.88	(1.71-2.07)	1.14E-25
HSPA1A	0.049429	0.561097	1.52	(1.11-2.27)	0.008464	1.01	(-1.25-1.28)	0.939835
